# Discrepancies in the Reactivity of Mesorectal versus Lateral Lymph Nodes Post-Neoadjuvant Chemoradiotherapy for Rectal Cancer: Insights from Radiological and Pathological Perspectives

**DOI:** 10.1245/s10434-025-17777-w

**Published:** 2025-07-10

**Authors:** Zixuan Zhuang, Xuyang Yang, Yang Zhang, Xiangbing Deng, Mingtian Wei, Ziqiang Wang

**Affiliations:** Colorectal Cancer Center, Department of General Surgery, West China Hospital, Sichuan University, Chengdu, Sichuan Province China

**Keywords:** Rectal cancer, Neoadjuvant chemoradiotherapy, Lateral lymph nodes, Mesorectal lymph nodes, Lymph node regression grade, Prognosis

## Abstract

**Introduction:**

The heterogeneous treatment response of lateral lymph nodes (LLN) and mesorectal lymph nodes (MLN) to neoadjuvant chemoradiotherapy (NCRT) in locally advanced rectal cancer (LARC) remains poorly understood. This study investigates imaging regression patterns, pathological lymph node regression grade (LRG), and prognostic implications in patients with LARC undergoing radical resection and lateral lymph node dissection (LLND) after NCRT.

**Patients and Methods:**

A total of 110 patients with LARC undergoing radical resection and LLND after NCRT (2018–2024) were enrolled. Visible MLNs and LLNs on initial magnetic resonance imaging (MRI) were matched node-by-node on post-NCRT MRI, and short-axis (SA) diameter regression was calculated. Pathological LRG scores, including LRG-max, LRG-sum, and LRG-ratio, were assessed on the basis of tumor cell proportion and fibrosis. Differences in radiological and pathological regression between MLNs and LLNs were analyzed at lymph node (LN) and patient levels.

**Results:**

MRI assessed 1744 LNs (811 MLNs, 933 LLNs pre-NCRT; 546 MLNs, 969 LLNs post-NCRT). MLNs exhibited higher disappearance rates (32.7% versus 2.4%, *P* < 0.0001) and greater SA regression (0.596 ± 0.333 versus 0.214 ± 0.242, *P* < 0.0001) than LLNs. Histopathological re-evaluation of 2108 LNs (916 MLNs, 1192 LLNs) revealed LLNs had higher LRG-max (37.13 versus 21.37, *P* = 0.011) and LRG-ratio (3.45 ± 1.29 versus 2.47 ± 0.96, *P* = 0.0003). LRG-sum was an independent prognostic factor for both MLNs and LLNs.

**Conclusions:**

LLNs demonstrated lower imaging regression, higher pathological residual cancer, and poorer treatment response compared with MLNs, indicating greater NCRT tolerance.

**Supplementary Information:**

The online version contains supplementary material available at 10.1245/s10434-025-17777-w.

Neoadjuvant chemoradiotherapy (NCRT) is a crucial component of the comprehensive treatment for patients with stage II/III rectal cancer. Although NCRT has not substantially increased overall survival compared with total mesorectal excision (TME) alone, it has notably reduced the rate of central recurrence caused by mesorectal cancer remnants, with recurrence rates dropping from 4–27% to 2.4–11%.^1–3^ Meanwhile, lateral pelvic recurrence resulting from lateral lymph node metastasis (LLNM) has emerged as the main pattern of local recurrence, accounting for up to 82.7% of cases.^4,5^

Previous studies have demonstrated that the probability of complete regression of enlarged lateral lymph nodes as observed on imaging after NCRT is extremely low. Only approximately 5% of cases show total disappearance of these lymph nodes.^6,7^ Moreover, existing evidence suggests that mesorectal lymph nodes (MLNs) and lateral lymph nodes (LLNs) may respond differently to NCRT, as indicated by lymph node metastasis diagnosis and prognosis.^8,9^ Nevertheless, studies that specifically examine the disparities in their imaging regression behaviors are scarce. This lack of research complicates the formulation of individualized treatment strategies, especially when making decisions about adopting lateral lymph node dissection (LLND) or a “watch-and-wait” approach for patients with pretreatment-suspected LLNM.

Tumor regression grade (TRG), defined by the ratio of fibrosis to residual cancer cells after NCRT, is a key indicator of primary tumor response and an important prognostic factor for patients with LARC.^10,11^ NCRT also reduces lymph node retrieval rates and causes downstaging of the N stage. However, so far, there is limited information about the residual tumor burden within the lymph nodes after NCRT. Histological regression in MLNs, characterized by features such as mucinous deposits, has been observed after NCRT.^12,13^ Caricato et al. first reported lymph node regression in MLNs.^14^ However, MLNs and LLNs might exhibit different regression patterns. Therefore, assessing the lymph node regression grade (LRG) for both MLNs and LLNs is of utmost importance, as their treatment responses can have significant prognostic implications.

In this study, we initially investigated the radiological regression patterns of MLNs and LLNs in patients with LARC after NCRT. On the basis of this, we assessed the LRG of metastatic MLNs and LLNs, described their treatment response patterns, and analyzed their potential prognostic value.

## Patients and Methods

### Study Population

**Fig. 1:**
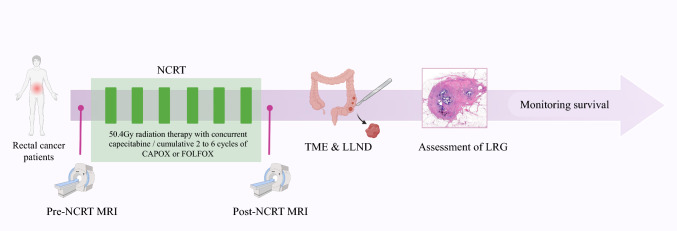
The workflow in this study

This cohort analysis encompassed 110 patients diagnosed with LARC who underwent NCRT followed by TME and LLND between January 2018 and January 2024. The inclusion criteria were as follows: (1) rectal cancer located within ≤ 10 cm from the anal margin; (2) patients who received standard long-course NCRT regimen; (3) radiation fields covering the mesorectum, internal iliac, obturator, and external iliac regions, with all regions receiving a uniform dose of 45–54 Gy; (4) both pre- and post-NCRT magnetic resonance imaging (MRI) data were available for analysis; and (5) presence of both MLNs and LLNs with a short-axis diameter ≥ 3 mm on initial MRI. The exclusion criteria were as follows: (1) absence of complete imaging or clinical data and (2) recurrent rectal cancer. The overall workflow is illustrated in Fig. 1, with a detailed breakdown of the steps provided below.

### Preoperative Chemoradiotherapy and Surgery

Patients with LARC were recommended to receive neoadjuvant therapy according to European Society for Medical Oncology (ESMO) guidelines.^15^ In this study, the preoperative treatment regimens included standard long-course chemoradiotherapy (CRT), consisting of 50.4 Gy delivered in 25–28 fractions with concurrent capecitabine. A subset of patients additionally received two to six cycles of induction and/or consolidation CAPOX chemotherapy (total neoadjuvant therapy (TNT) strategy), comprising oxaliplatin and capecitabine. Surgical resection was scheduled 6–8 weeks after the completion of NCRT. Radical surgical resection included TME and LLND. LLND was indicated when pretreatment imaging revealed a large LLN with a short-axis diameter > 5 mm that exhibited no significant regression following NCRT. Bilateral LLND was considered only when clinically suspected LLNM was identified before treatment on both sides. Postoperative adjuvant chemotherapy was recommended for patients who presented with stage III disease or stage II with high-risk factors, as determined by the pathological findings. Patients who underwent TNT regimen did not receive any postoperative adjuvant chemotherapy.

### Magnetic Resonance (MR) Imaging Protocol

Magnetic resonance imaging was performed with a 3T MAGNETOM Skyra MR scanner (Siemens Healthineers, Malvern, PA, USA) employing an 18-channel body coil. All patients were given an intravenous antiperistaltic agent (10 mg of raniscopolamine hydrochloride) 30 min before MRI for bowel preparation. The high-resolution rectal MRI protocol included turbo spin-echo sagittal, oblique coronal, and oblique axial T2-weighted imaging (WI), as well as diffusion-weighted imaging (DWI). The parameters for the oblique axial T2-weighted imaging sequence were set as follows: repetition time/echo time (TR/TE) of 6890/100 ms; slice thickness of 3 mm; voxel size of 0.3 mm × 0.3 mm × 3 mm; field of view (FOV) of 180 mm; matrix of 384 × 346; 48 slices; three averages; and a total scanning time of 5 min and 5 s. The parallel acquisition technique employed was the generalized autocalibrating partial parallel acquisition (GRAPPA) acceleration factor. For the oblique axial DWI sequence, a transverse echo-planar imaging diffusion sequence was used, with the highest *b* value set at 1000 s/mm^2^. The DWI parameters, including FOV, slice thickness, and gap, were matched to those of the oblique axial T2-weighted imaging to ensure accurate tumor localization. The total scan time for the entire protocol was 30 min.

### Radiological Evaluation

All rectal MRI images of patients with rectal cancer were independently analyzed by two experienced radiologists, both blinded to the clinicopathological data. High-resolution oblique-axis T2-weighted MRI sequences were used for evaluation. Any discrepancies in radiological conclusions were resolved through discussion to reach a consensus.

The classification of lateral lymph node regions was consistent with our previous research and adhered to the latest guidelines from the Japanese Society for Cancer of the Colon and Rectum (JSCCR), with modifications for clinical practicality.^16,17^ As illustrated in Supplementary Fig. S1, obturator lymph nodes were subdivided into cranial and caudal obturator LNs. Similarly, internal iliac LNs were further categorized into three groups: proximal internal iliac LNs, distal internal iliac LNs, and extended distal internal iliac LNs. The specific definitions of each LLN region are as follows: Common iliac LNs: LNs located along the common iliac artery, from the aortic bifurcation to the bifurcation of the common iliac artery. External iliac LNs: LNs distributed along the external iliac artery, spanning from the branching point of the external and internal iliac arteries to the transition of the external iliac artery into the femoral artery. Internal iliac LNs: LNs located along the internal iliac artery, extending from its origin at the bifurcation of the common iliac artery to its distal continuation beyond the pelvic cavity. Proximal internal iliac LNs: LNs distributed from the bifurcation of the common iliac artery to the origin of the superior vesical artery. Distal internal iliac LNs: LNs located from the origin of the superior vesical artery to the plane where the piriformis muscle disappears. Extended distal internal iliac LNs: LNs distributed beyond the plane of the piriformis muscle, in the extended region of the internal iliac artery. Obturator LNs: LNs located within the region defined by the lateral border of the internal iliac artery, the medial border of the external iliac artery, the obturator internus muscle, and the psoas major muscle. Obturator cranial LNs: LNs located cranial to the obturator artery within the obturator region. Obturator caudal LNs: LNs located caudal to the obturator artery within the obturator region.

The imaging data of patients before and after NCRT were retrospectively reviewed. Post-NCRT MRI was typically performed 4–7 weeks after completion of radiotherapy. All measurable MLNs and LLNs were meticulously identified with close reference to the radiotherapy field. The short-axis (SA) diameter of each LN was measured at its largest cross-sectional level, and its region was annotated. Relatively fixed anatomical structures, such as blood vessels, muscle boundaries, and bony landmarks, were used as reference points to locate corresponding LNs on post-NCRT MRI. For each LN, the SA diameter, regional classification, and correspondence between pre- and post-treatment measurements were recorded at the largest cross-sectional level on MRI (Supplementary Fig. S2). LNs with an SA diameter < 3 mm on the initial MRI-T2WI sequence were excluded from the analysis owing to their small size or volume effects that rendered measurement unreliable. In this study, the post-NCRT short-axis (SA) regression rate of each LN was calculated as follows: SA regression rate (%) = (pre-treatment SA − post-treatment SA) / pre-treatment SA × 100%. On the basis of the Response Evaluation Criteria in Solid Tumors (RECIST) version 1.1,^18^ the post-treatment changes in MLNs and LLNs were defined as follows: disappearance: LNs that were no longer detectable on post-NCRT MRI, with a regression rate of 100%. Shrinkage: LNs with a post-treatment SA regression rate of ≥ 30%, indicating at least a 30% decrease in SA. Stable: LNs with a post-treatment SA regression rate between −20% and 30%, indicating a decrease of less than 30% or an increase of less than 20% in SA. Progressive: LNs with a post-treatment SA regression rate of ≤ −20%, indicating at least a 20% increase in SA.

### Pathologic Examination

Routine hematoxylin and eosin (H&E) staining was performed on all LNs. The pathological tumor regression grade was determined according to the American Joint Committee on Cancer (AJCC) TRG classification,^19^ defined as follows: TRG 0, no residual tumor cells; TRG 1, single cells or small clusters of tumor cells; TRG 2, residual tumor with desmoplastic response; and TRG 3, minimal evidence of tumor response. The regression level of metastatic LNs in response to NCRT was assessed on the basis of the proportion of tumor cells and fibrosis and classified using a six-tier grading system: LRG 0, normal lymph node architecture with no evidence of regression or residual cancer cells; LRG 1, 100% fibrosis; LRG 2, < 25% remaining cancer cells; LRG 3, 25–50% scattered glandular elements with fibrosis; LRG 4, > 50% viable cancer cells; and LRG 5, complete replacement by cancer cells. Representative images of each LRG are provided in Supplementary Fig. S3. Given that each patient had a variable number of LNs and that individual LNs could exhibit different regression grades depending on their response to treatment, we calculated three parameters: the maximum LRG (LRG-max), the sum of the LRG scores across all metastatic LNs (LRG-sum), and the LRG-ratio (LRG sum divided by the total number of positive lymph nodes). The TRG and LRG assessments for all specimens were conducted by two pathologists specializing in gastrointestinal pathology. Both pathologists jointly reviewed all specimens and reached a consensus through discussion.

### Follow-Up

Physical examinations, including digital rectal examinations and serum tumor marker assessments, were conducted every 3 months during the first 2 years, every 6 months in the subsequent 3 years, and annually thereafter. Chest computed tomography (CT) and enhanced abdominal CT scans were recommended biannually for the first 2 years and annually thereafter. Local recurrence (LR) was defined as tumor recurrence occurring in the anastomotic site, pelvic cavity, or perineum. Lateral local recurrences (LLR) were defined as tumor regrowth within any of the LLN basins previously identified. Overall survival was defined as the interval between the date of surgery and death or the last follow-up. Disease-free survival was defined as the period from surgery to the detection of either local or distant recurrence.

### Statistical Analysis

Statistical analyses were performed using R 4.0.5 software (http://www.rproject.org), SPSS 23.0 software (SPSS INC., Chicago, USA), and GraphPad Prism 10 (GraphPad Software, La Jolla, CA, USA). Continuous variables were presented as mean ± standard deviation (SD), and differences between the two groups were assessed using Student’s *t*-test. Categorical variables were expressed as absolute numbers with percentages, and differences were evaluated using Pearson’s chi-squared test or Fisher’s exact test, as appropriate. Optimal cutoff values for LRG-sum were determined using the X-tile program (http://www.tissuearray.org/rimmlab/).^20^ Overall survival and disease-free survival were analyzed using the Kaplan–Meier method, with comparisons between groups assessed by the log-rank test. A *p*-value < 0.05 was considered statistically significant.

## Results

### Clinicopathological Characteristics of Patients

In this study, 110 patients with rectal adenocarcinoma were analyzed, comprising 63 males (57.3%) and 47 females (42.7%). The mean distance of the tumor from the anal verge was 4.1 cm. Clinically, the majority of patients presented with T3 (55.5%) and N2 (51.8%) tumors. Of the entire cohort, 51 patients (46.4%) received standard long-course chemoradiotherapy, whereas 59 patients (53.6%) underwent total neoadjuvant therapy. After NCRT, 22 patients (20.0%) achieved a pathological complete response, with ypT3 (42.7%) and ypN0 (55.5%) being the most common pathological stages. Among the 49 patients with pathological lymph node metastases, 9 (18.4%) had metastasis confined to MLNs, 14 (28.5%) had isolated involvement of LLNs, and 26 (53.1%) had metastases in both MLNs and LLNs. A total of 2108 lymph nodes were harvested during histopathological examination, including 916 MLNs and 1192 LLNs, with 80 metastatic MLNs and 71 metastatic LLNs identified. The mean lymph node ratio (LNR) was 0.08 ± 0.18. The median follow-up period was 39 months. Detailed clinicopathological characteristics are presented in Table 1.

**Table 1:** Clinicopathological characteristics of patients

Characteristics	Value
Age (years)	56 (22–80)
*Gender*	
Male	63 (57.3)
Female	47 (42.7)
Median follow-up time, Months	39 (4–96)
Distance of the tumor	4.1 ± 1.9
BMI (kg/m^2^)	23.65 ± 3.48
CEA	9.36 ± 18.77
*cT*	
T2	1 (0.9)
T3	61 (55.5)
T4	48 (43.6)
*cN*	
N0	0 (0)
N1	53 (48.2)
N2	57 (51.8)
Tumor diameter (cm)	2.6 ± 1.9
*Neoadjuvant therapy regimens*	
TNT	59 (53.6)
CRT	51 (46.4)
Tumor diameter (cm)	2.6 ± 1.9
*Lymphovascular invasion*	
Negative	100 (90.9)
Positive	10 (9.1)
Perineural invasion	
Negative	86 (78.2)
Positive	24 (21.8)
Lymph node ratio	0.08 ± 0.15
*CRM*	
Negative	104 (94.5)
Positive	6 (5.5)
*ypT stage*	
T0	22 (20.0)
T1	2 (1.8)
T2	35 (31.8)
T3	47 (42.7)
T4	4 (3.6)
*ypN stage*	
N0	61 (55.5)
N1	38 (34.5)
N2	11 (10.0)
*Tumor regression grade*	
Grade 0	22 (20.0)
Grade 1	17 (15.5)
Grade 2	60 (54.5)
Grade 3	11 (10.0)
*Number of ypN-positive disease*	
MLN positive only	9 (18.4)
LLN positive only	14 (28.5)
MLN and LLN positive	26 (53.1)
*Number of retrieved lymph nodes*	
MLN	916
LNN	1192
*Number of positive lymph nodes*	
MLN	80
LLN	71

*BMI* body mass index, *CEA* carcinoembryonic antigen, *CRM* circumferential resection margin, *MLN* mesorectal lymph nodes, *LLN* lateral pelvic lymph nodes

### Association Between MLN and LLN Regression Rate in Radiological Evaluation

**Fig. 2:**
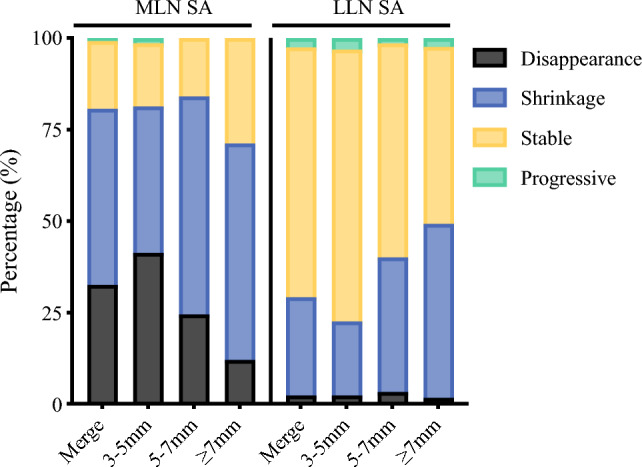
Radiologic response patterns of MLNs and LLNs stratified by short-axis diameter

A total of 811 MLNs were measured on pretreatment MRI-T2WI. Among these, 476 (58.7%) had an SA diameter between 3 and 5 mm, 220 (27.1%) had an SA diameter between 5 and 7 mm, and 115 (14.2%) had an SA diameter ≥ 7mm. Of the total MLNs, 264 (32.6%) were located below the peritoneal reflection, and 547 (67.4%) were located above the peritoneal reflection. After NCRT, 265 (32.7%) MLNs showed complete disappearance on imaging, 389 (48%) exhibited shrinkage, 151 (18.6%) remained stable, and 6 (0.7%) showed enlargement (Table 2, Supplementary Table S1). Notably, both the likelihood of disappearance and the SA regression rate decreased progressively with initial SA diameters of 3–5 mm, 5–7 mm, and ≥ 7 mm, with significant differences observed between these groups (Fig. 2, Table 2, Supplementary Table S2).

**Table 2:** Radiological and pathological characteristics of MLN and LLN

	Characteristics	MLN	LLN	*P*-value
Radiological characteristics	No. (%)	811	993	
	3–5 mm	476 (58.7)	690 (69.5)	
	5–7 mm	220 (27.1)	175 (17.6)	
	≥ 7 mm	115 (14.2)	129 (13.0)	
	Disappearance	265 (32.7)	24 (2.4)	
	Shrinkage	389 (48)	265 (26.7)	
	Stable	151 (18.6)	679 (68.4)	
	Progressive	6 (0.7)	25 (2.5)	
	Regression rate, (mean ± SD)	0.596 ± 0.333	0.214 ± 0.242	< 0.001
	3–5 mm	0.633 ± 0.351	0.175 ± 0.230	< 0.001
	5–7 mm	0.581 ± 0.293	0.279 ± 0.238	< 0.001
	≥ 7 mm	0.474 ± 0.302	0.334 ± 0.253	< 0.001
Pathological characteristics	No. (%)	916	1192	
	LRG0	818 (89.3)	1104 (92.6)	
	LRG1	24 (2.6)	17 (1.4)	
	LRG2	43 (4.7)	12 (1)	
	LRG3	23 (2.3)	20 (1.8)	
	LRG4	13 (1)	29 (2.4)	
	LRG5	1 (0.1)	10 (0.8)	
	LRG-max, (mean rank)	21.37	31.63	0.011
	LRG-ratio, (mean ± SD)	2.47 ± 0.96	3.45 ± 1.29	< 0.001

*MLN* mesorectal lymph nodes, *LLN* lateral pelvic lymph nodes, *LRG* lymph node regression grade, *LRG-max* maximum tumor regression grade in lymph node, *LRG-ratio* LRG-sum divided by the number of positive lymph nodes

During the initial MRI-T2WI assessment, a total of 993 LLNs were measured. Among these, 690 (69.5%) had an SA diameter between 3 mm and 5 mm, 175 (17.6%) between 5 and 7 mm, and 129 (13%) had an SA diameter ≥ 7 mm. The regional distribution of these LLNs was as follows: 220 (22.2%) in the common iliac region, 126 (12.7%) in the external iliac region, 163 (16.4%) in the internal iliac region, and 484 (48.7%) in the obturator region. After NCRT, only 24 (2.4%) LLNs completely disappeared, 265 (26.7%) exhibited shrinkage, 679 (68.4%) remained stable, and 25 (2.5%) showed progression (Table 2, Supplementary Table S3). Notably, compartment-specific analysis revealed significant heterogeneity in post-treatment nodal regression probabilities across lateral pelvic regions (*P* < 0.001), with the internal iliac and obturator compartments demonstrating the highest regression probability (Supplementary Fig. S4). The overall regression rate for LLNs was 0.214 ± 0.242, with the highest rates observed in the internal iliac and obturator regions (*P* < 0.001). In addition, the post-NCRT regression rate increased progressively with initial SA diameters of 3–5 mm, 5–7 mm, and ≥ 7 mm (*P* < 0.001) (Fig 2, Table 2, Supplementary Table S4).

When comparing MLNs and LLNs, the initial MRI-T2WI revealed a greater number of LLNs than MLNs (993 versus 811). However, the proportion of LLNs with an SA diameter ≥ 5 mm was lower compared with MLNs (30.6% versus 41.3%). Following NCRT, MLNs demonstrated a significantly higher regression rate (0.596 ± 0.333 versus 0.214 ± 0.242, *P* < 0.001) and disappearance rate (32.7% versus 2.4%, *P* < 0.001) compared with LLNs (Table 2, Fig. 2).

### Association Between MLN and LLN in Pathological Evaluation

**Fig. 3:**
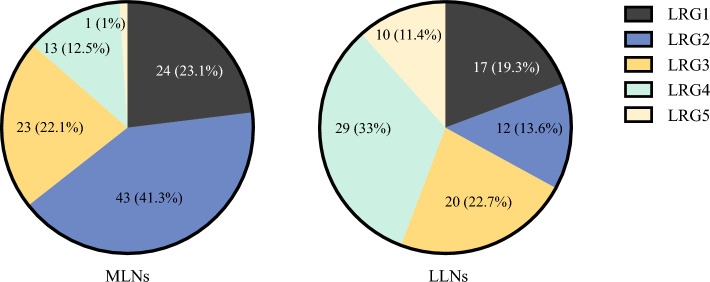
Lymph node regression grade distribution of MLNs and LLNs; **A** MLNs; **B** LLNs

**Fig. 4:**
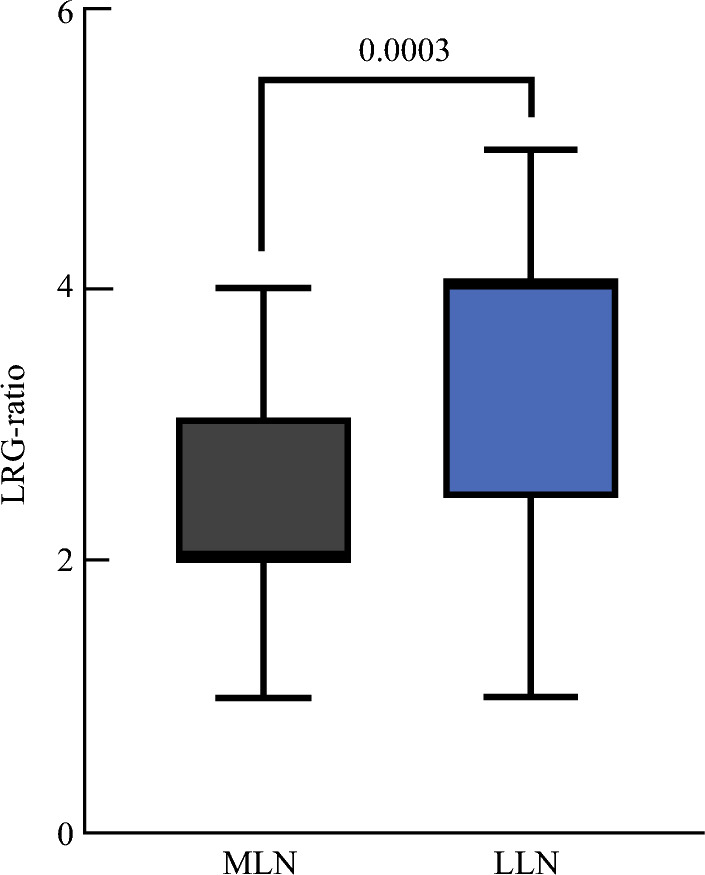
Comparison of LRG-ratio between MLNs and LLNs

A total of 2108 LNs were harvested, including 916 MLNs and 1192 LLNs. The LRG distributions of the harvested MLNs were as follows: LRG0 in 818 LNs (89.3%), LRG1 in 24 LNs (2.6%), LRG2 in 43 LNs (4.7%), LRG3 in 23 LNs (2.3%), LRG4 in 13 LNs (1.4%), and LRG5 in 1 LN (0.1%). The LRG distributions of the harvested LLNs were as follows: LRG0 in 1104 LNs (92.6%), LRG1 in 17 LNs (1.4%), LRG2 in 12 LNs (1.0%), LRG3 in 20 LNs (1.8%), LRG4 in 29 LNs (2.4%), and LRG5 in 10 LNs (0.8%) (Fig 3, Table 2). A subgroup analysis of 26 patients with both MLN and LLN metastases was conducted to evaluate differences in the LRG-max and LRG-ratio (Supplementary Table S5). The distribution of LRG-max is shown in Supplementary Fig. S5. Among MLNs, the most common LRG-max score was 2 (38.5%), whereas among LLNs, the most frequent LRG-max score was 4 (46.2%). The mean rank of LRG-max in LLNs was 37.13, significantly higher than the mean rank of 21.37 in MLNs (*Z* = 2.529, *P* = 0.011). Fig. 4 illustrates the differences in LRG-ratio between MLNs and LLNs, with LLNs showing a significantly higher LRG-ratio (3.45 ± 1.29) compared with MLNs (2.47 ± 0.96) (*P* < 0.001).

### Association Between LRG and Clinicopathological Characteristics

Among the 110 included patients, 49 had lymph node metastases. The average LRG-sum for MLNs was 2.19 ± 4.93, while for LLNs, it was 2.43 ± 4.43. As shown in Supplementary Fig. S6, the LRG-sum was closely associated with TRG, with higher LRG-sum significantly correlated with higher TRG levels (all *P* < 0.05). In addition, the LRG-sum increased significantly with advancing ypN stage (all *P* < 0.0001). Consequently, for both MLNs and LLNs, higher LRG-sums were strongly associated with higher TRG levels and more advanced ypN stages (Supplementary Fig. S7).

### Prognostic Value of LRG

After a median follow-up of 39 months, 28 patients (25.5%) died, and 40 patients (36.4%) experienced tumor recurrence or metastasis, including 5 (4.5%) with local recurrence (3 LLR) and 40 (36.4%) with distant metastases. Utilizing the X-tile program, the optimal cutoff points for LRG-sum were determined as 1 and 4 (Supplementary Fig. S8). On the basis of these thresholds, the patient cohort was stratified into the following groups: for MLN LRG-sum, a low group (≤ 1, *n* = 74), an intermediate group (2–4, *n* = 23), and a high group (≥ 5, *n* = 13); and for LLN LRG-sum, a low group (≤ 1, *n* = 149), an intermediate group (2–4, *n* = 27), and a high group (≥ 5, *n* = 17). As illustrated in Supplementary Figs. S9 and S10, higher LRG-sum scores were associated with worse overall survival (OS). The 3-year OS rates for the MLN-LRG low, intermediate, and high groups were 84.2%, 62.1%, and 38.1%, respectively (*P* < 0.001). Similarly, the 3-year OS rates for the LLN-LRG low, intermediate, and high groups were 90.2%, 58.8%, and 54.1%, respectively (*P* < 0.001). In addition, higher LRG-sum scores were also associated with worse disease-free survival (DFS). The 3-year DFS rates for the MLN-LRG low, intermediate, and high groups were 70.9%, 58.2%, and 22.0%, respectively (*P* < 0.001). For the LLN-LRG low, intermediate, and high groups, the 3-year DFS rates were 78.2%, 44.8%, and 29.0%, respectively (*P* < 0.001) (Supplementary Table S6).

We further evaluated the prognostic significance of LRG-sum for DFS. Univariate analysis indicated that several factors, including lymph node ratio, ypN stage, TRG, number of positive MLNs and LLNs, MLN LRG-max, LLN LRG-max, MLN LRG-sum, and LLN LRG-sum were all independently associated with DFS (Supplementary Table S7). Cox regression analysis confirmed that MLN LRG-max (hazard ratio (HR) = 0.513, 95% confidence interval (CI) 0.285–0.924, *P* = 0.026), MLN LRG-sum (HR = 1.131, 95% CI 0.929–1.378, *P* = 0.041), and LLN LRG-sum (HR = 0.907, 95% CI 0.695–1.185, *P* = 0.033) were independent prognostic factors for DFS following NCRT. Among these, LRG-sum emerged as one of the most significant predictors (Supplementary Fig. S11).

## Discussion

To the best of our knowledge, this is the first study to comprehensively investigate the regression patterns of MLNs and LLNs following NCRT for LARC. By integrating radiological and pathological analyses, we clarify the differences in treatment responses, highlight their prognostic significance, and underscore their potential clinical implications.

Radiological analysis revealed that MLNs with larger initial SA diameters had lower disappearance and regression rates after NCRT. Previous studies reported a significant reduction in the pathological yield of MLNs after NCRT compared with untreated patients, with a smaller decrease in positive MLNs compared with negative MLNs.^21^ Mechera et al. found that, on average, 3.9 fewer MLNs were pathologically detected post-NCRT, including a reduction of 0.7 positive MLNs, suggesting that benign MLNs are more likely to regress than malignant ones.^22^ However, owing to the lack of node-by-node correspondence between imaging and pathological findings in this study, we cannot confirm whether larger and malignant MLNs are more likely to retain pathological cancer residue post-treatment. Nevertheless, previous studies have shown that larger LNs are more likely to be malignant and that poorly regressed LNs are more likely to retain pathological cancer residue,^23,24^ suggesting greater resistance to NCRT in larger and malignant MLNs. Radiological analysis of LLNs revealed that the internal iliac and obturator regions exhibited the highest regression rates, with larger initial SA diameters correlating with greater regression post-treatment. Yamaoka et al. reported that the obturator region contained the most LLNs overall, while the internal iliac region had a higher proportion of malignant LLNs, regardless of NCRT administration.^25^ Similarly, Ogura et al. observed region-specific LLN recurrence rates following NCRT.^26^ Despite consistent radiation doses across the lateral pelvic regions in this study, the varying degrees of radiological regression suggest potential biological differences among LLNs in different regions.

Comparison of MLNs and LLNs revealed that, regardless of initial SA size, LLNs had significantly lower disappearance rates and regression rates after NCRT. Larger initial SAs in MLNs and LLNs have been associated with higher rates of pathological metastases.^27,28^ While an SA ≥ 5 mm is widely used as the optimal cutoff for diagnosing MLN metastases, substantial overlap between benign and malignant MLNs limits its diagnostic accuracy.^23^ In contrast, SA has higher diagnostic accuracy for LLN metastases, with several multicenter studies and international consensus identifying 7 mm on pretreatment MRI as the optimal cutoff for defining metastatic lateral lymph nodes.^6,26,29,30^ These findings highlight potential biological differences between MLNs and LLNs. Notably, we observed that as SA increased, the difference in regression rates between MLNs and LLNs diminished, although it remained statistically significant. As Kim et al. reported, increasing initial SA is associated with significantly higher rates of pathological cancer residue post-NCRT for both MLNs and LLNs.^27,28^ This suggests that resistance to NCRT may increase with larger initial SA, reducing disappearance rates and narrowing the disparity between MLNs and LLNs.

Studies indicate that up to 17% of LNs may retain residual tumor cells even when the primary tumor achieves a complete response.^31^ Predicting residual cancer in LNs after NCRT is thus crucial for assessing pathological complete response and determining the feasibility of a “watch-and-wait” strategy. For patients with LARC with a near-complete response and no pathological MLN residue after NCRT, initial local excision may be considered, with further pathological evaluation determining the need for additional TME. In patients with suspected LLNM, studies have shown that the pathological positivity rate of LLNs remains as high as 34.1% even after TNT, suggesting that NCRT alone may offer limited control.^32^ In contrast, the addition of selective LLND following NCRT has been associated with lower lateral recurrence rates (2.9–15.0% versus 11–27%)^33^ and is increasingly recognized as a preferred strategy for managing clinically evident LLNM. These findings emphasize the importance of evaluating the pathological status of MLNs and LLNs after NCRT. However, currently all research on LRG has focused primarily on MLNs, with limited exploration of LLNs. Pathological evaluation in this study revealed distinct differences in LRG between MLNs and LLNs. While the majority of MLNs (89.3%) and LLNs (92.6%) were classified as LRG0, higher-grade regression scores (LRG4 and LRG5), which reflect substantial residual tumor burden, were more frequently observed in LLNs (3.2%) than in MLNs (1.5%). In 2014, Mirbagheri et al. refined the LRG system on the basis of the relationship between residual cancer cells and regressive fibrosis.^11^ They also first introduced LRG-max and LRG-sum to quantify the worst LN score and the total tumor burden, respectively. In our analysis of patients with both MLN and LLN metastases, LLNs demonstrated significantly higher LRG-max (*P* = 0.011) and LRG-ratio (*P* < 0.001) compared with MLNs. This differential response is unlikely to be explained by baseline nodal size or radiation dosing, as imaging showed comparable nodal dimensions, and all patients received standardized radiation dosing. One possible explanation is that MLNs, as sentinel lymph nodes for rectal cancer, share a higher degree of homology with the primary tumor, making them more responsive to NCRT. Biological differences may also contribute; the lower incidence of fibrosis in LLNs might be associated with reduced immune cell infiltration within their microenvironment. Furthermore, it remains uncertain whether LLNs harbor a higher proportion of tumor cells expressing resistance-related genes. These observations, from the perspective of pathological tumor residue, further highlight the differential responsiveness of MLNs and LLNs to NCRT.

The study results reveal that the distribution of LRG-sum is strongly associated with the TRG for both MLNs and LLNs, with higher LRG scores significantly linked to more advanced ypN stages. As an essential prognostic marker after NCRT, TRG has been widely reported to correlate closely with LRG. Previous research has emphasized the relationship between LRG and complete primary tumor regression.^34,35^ Sun et al. reported that higher TRG grades were significantly associated with increased LRG scores, which were also linked to advanced ypT and ypN stages.^36^ Beppu et al. found that primary tumor radiosensitivity was associated with positive LNs.^37^ In addition, other studies have suggested that the degree of primary tumor response serves as a predictor of LN response; however, TRG alone is insufficient to reliably determine the presence of residual disease in LNs.^31^

The prognostic significance of MLN LRG in patients with LARC undergoing radical surgery after NCRT is well established.^38–40^ However, whether LLN LRG holds similar prognostic value in patients treated with TME and LLND after NCRT remains unclear. Using the X-tile program, we identified optimal cutoff values for MLN and LLN LRG-sum to stratify patients into prognostic groups. Higher MLN and LLN LRG-sum scores were significantly associated with worse OS and DFS, aligning with recent findings by Mirbagheri et al. and Sun et al.^12,36^ Multivariate Cox regression analysis further identified MLN LRG-max, MLN LRG-sum, and LLN LRG-sum as independent predictors of DFS. Similarly, a retrospective study by Cui et al. of 358 patients with LARC treated with NCRT highlighted LRG-sum, LRG-max, M-NLRG, and LRG as key independent prognostic factors for DFS.^40^ As LRG reflects both the degree of lymph node regression and the tumor burden before and after NCRT, it may offer greater prognostic value than traditional pathological risk factors.

This study has several limitations. First, as a single-center study, it is inherently prone to selection bias. Second, the sample size was small, and while the total number of pathologically examined LNs was relatively high, the number of malignant LNs was limited. Third, the lack of direct correlation between radiological and pathological findings at the individual node level hindered precise evaluation of tumor burden and regression for each lymph node. Lastly, owing to the relatively short median follow-up time, only a small number of local and lateral local recurrence events were observed, limiting the ability to perform reliable recurrence-related survival analyses.

## Conclusions

Our findings reveal distinct regression patterns between MLNs and LLNs after NCRT in patients with LARC. LLNs demonstrated a greater resistance to NCRT, manifested in a poorer treatment response and a higher residual tumor burden compared with MLNs. In addition, increased LRG scores were associated with more unfavorable prognoses. These results contribute to a more in-depth understanding of the differential responses of MLNs and LLNs to NCRT, potentially guiding more personalized treatment strategies for patients with LARC. 

## Supplementary Information

Below is the link to the electronic supplementary material.

Supplementary file1 (DOCX 6076 KB)

## Data Availability

The datasets obtained and/or analyzed during the current study are not publicly available due to confidentiality of patient information but are available from the corresponding author on reasonable request.
