# Supplementary material for: Discrepancies in the Reactivity of Mesorectal versus Lateral Lymph Nodes Post-Neoadjuvant Chemoradiotherapy for Rectal Cancer: Insights from Radiological and Pathological Perspectives
Source: Ann Surg Oncol. 2025 Jul 10;32(10):7293–303. doi: 10.1245/s10434-025-17777-w (PMC12454576; doi:10.1245/s10434-025-17777-w)
Supplement: Supplementary file 1 — Supplementary file1 (DOCX 6076 KB) [file 10434_2025_17777_MOESM1_ESM.docx]

**Supplements Figures**

**Fig. S1 The schematic diagram showed the seven lateral compartments. (I) (light red),** **common iliac compartment; (II) (sky blue),** **external iliac compartment; (III) (purple),** **obturator cranial compartment; (IV) (light orange),** **obturator caudal compartment; (V) (yellow):** **Proximal internal iliac compartment; (VI) (light green):** **Distal internal iliac compartment; (VII) (blue):** **Extended distal internal iliac compartment.**

**Figure adapted/reproduced from: Yang X et al., Cancer Medicine, 2024 Sep;13(18): e70170. © The Authors. Published by Wiley under a CC BY license.**


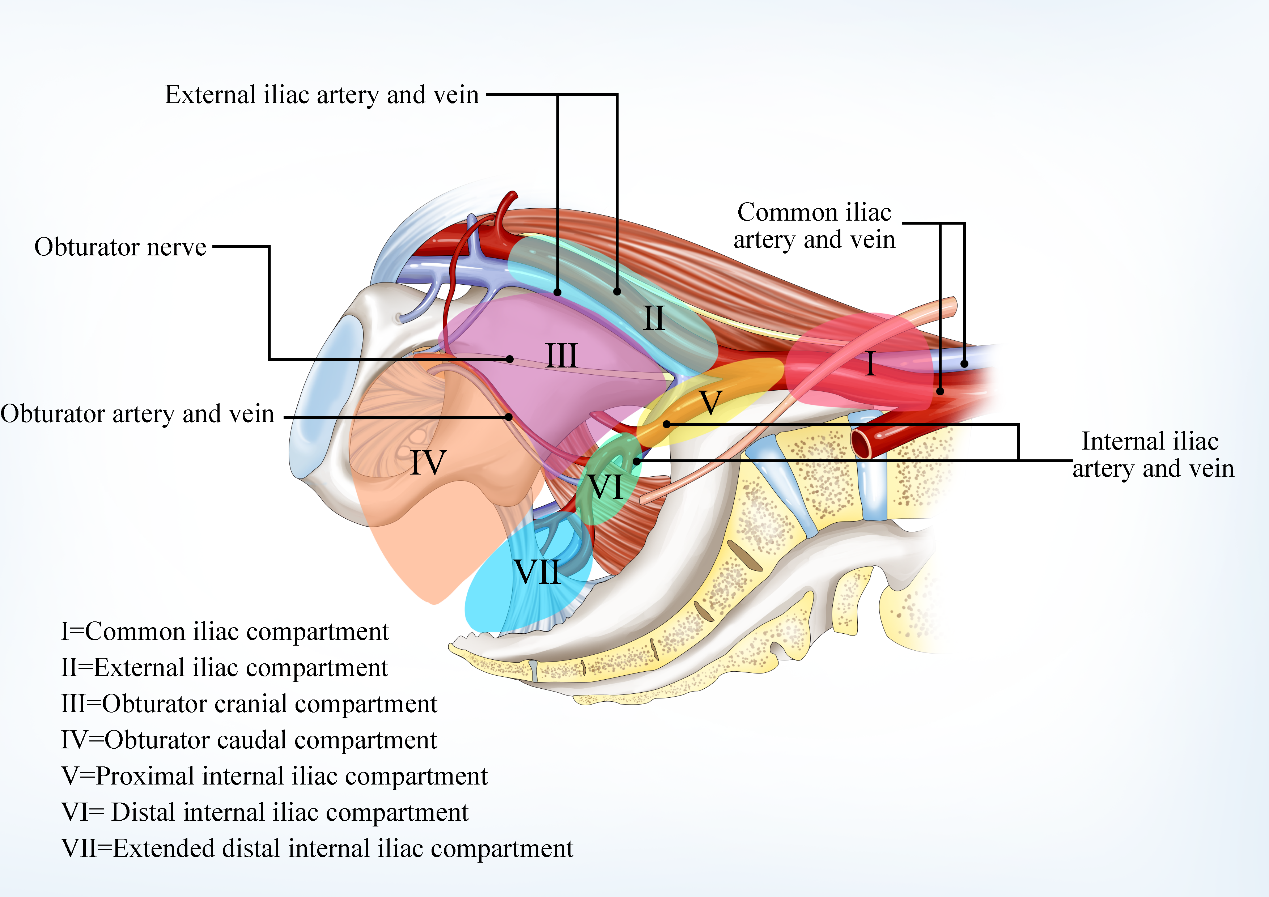


**Fig. S2 Illustration of node-by-node correspondence on MRI-T2WI before and after NCRT. a Pre-NCRT; b Post-NCRT. The yellow arrow indicates a right distal internal iliac lateral lymph node.**

**
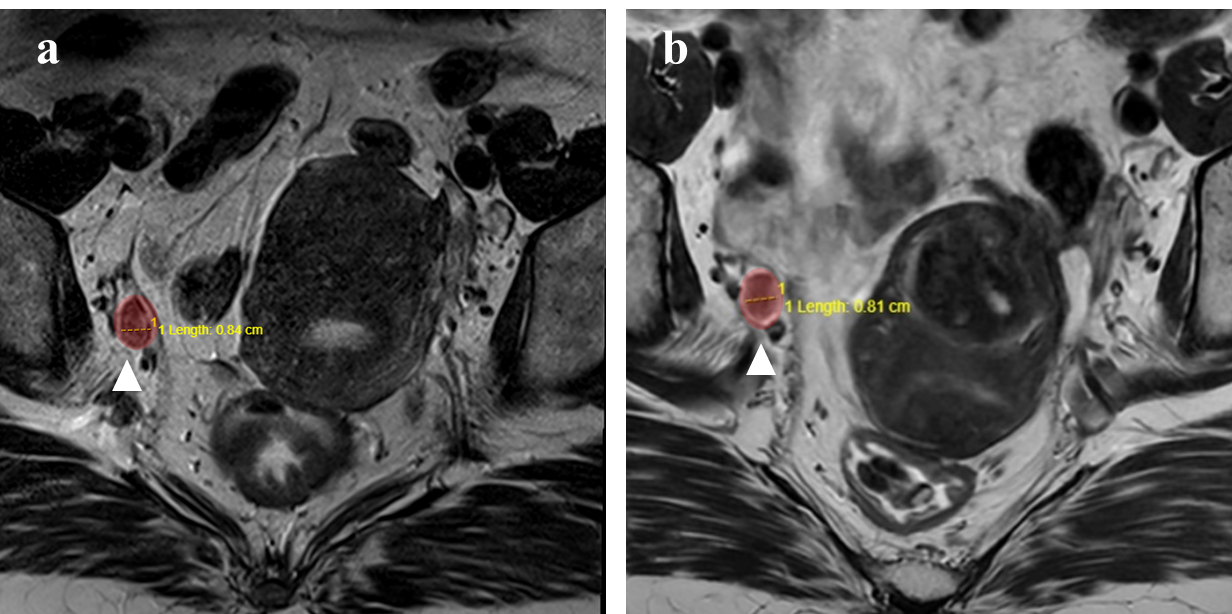
**

**Fig. S3 Representative images of each LRG score. a LRG 0; b LRG 1; c LRG 2; d LRG 3; e LRG 4; f LRG5.**


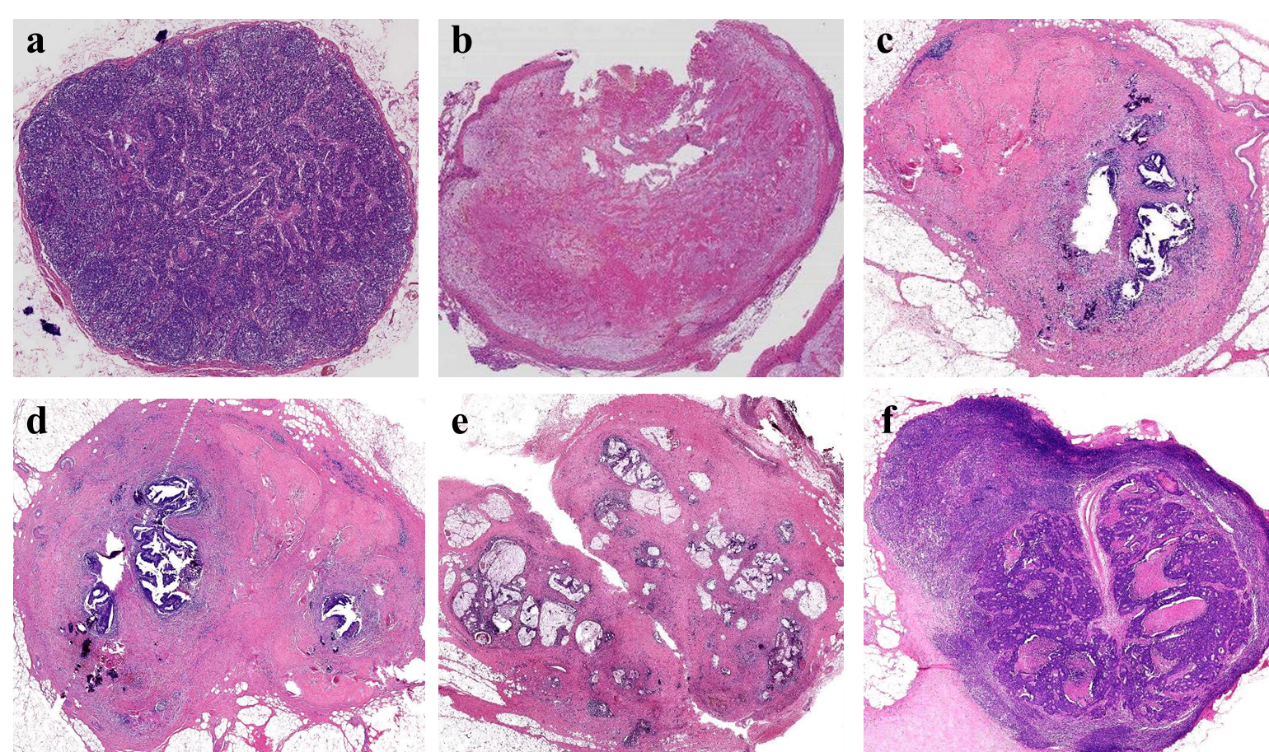


**Fig S4 Comparative of Treatment Response Across Lateral Lymph Node Compartments.**

**Fig. S5 Distribution of LRG-max Scores for MLNs and LLNs.**

**A. MLNs; B. LLNs**

**Fig. S6 LRG-sum stratified by TRG in MLNs and LLNs. (a) MLN; (b) LLN.**

**Fig. S7 LRG-sum stratified by ypN stage in MLNs and LLNs. (a) MLN; (b) LLN.**

**** ****

**Fig. S8 Cutoff points for LRG-sum determined by X-tile program.**


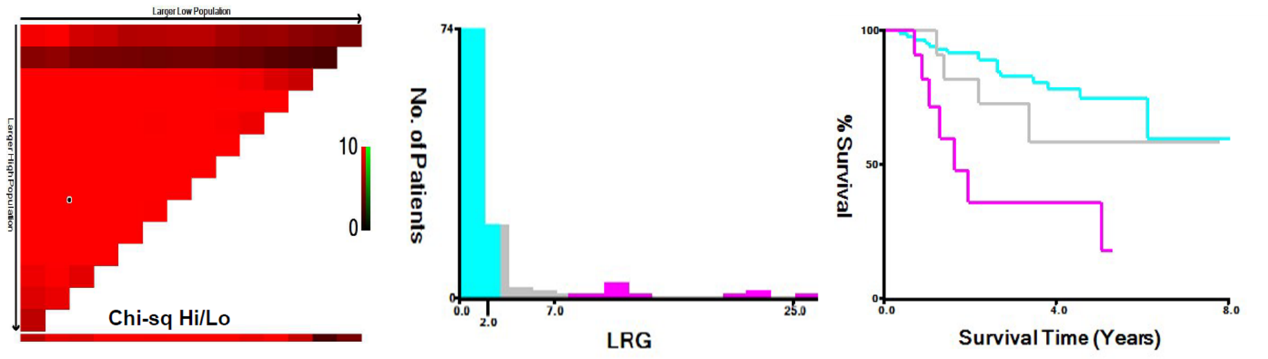


**Fig. S9 Overall survival stratified by the three LRG-sum groups. (a) MLN; (b) LLN.**

**a b**


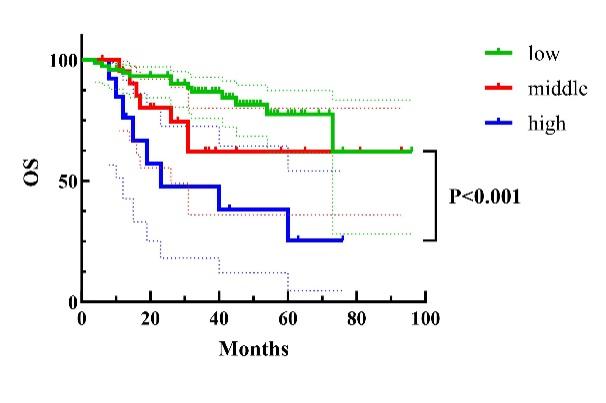

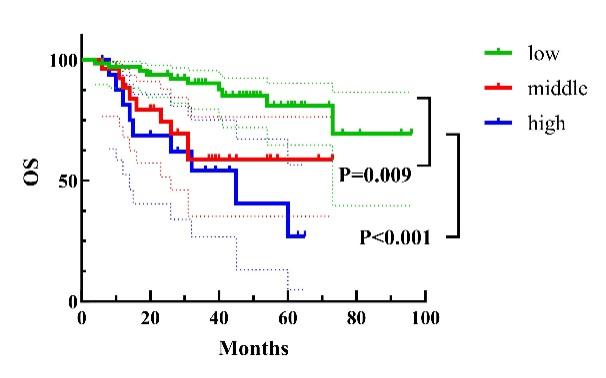


**Fig. S10 Disease-free survival outcomes between three LRG-sum groups. (a) MLN; (b) LLN.**

**a b**


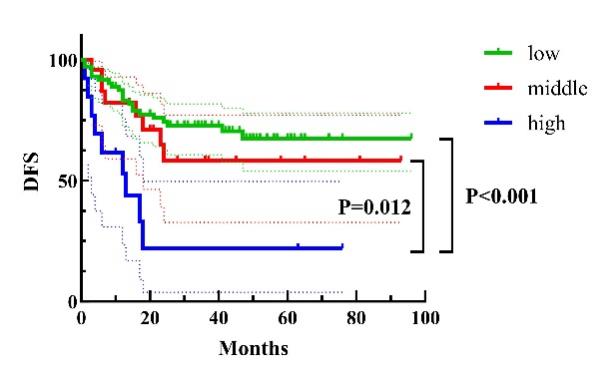

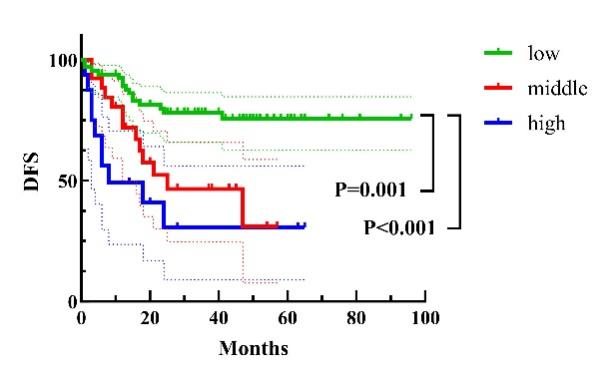


**Fig. S11 Forest plot of Cox regression analysis.**

**
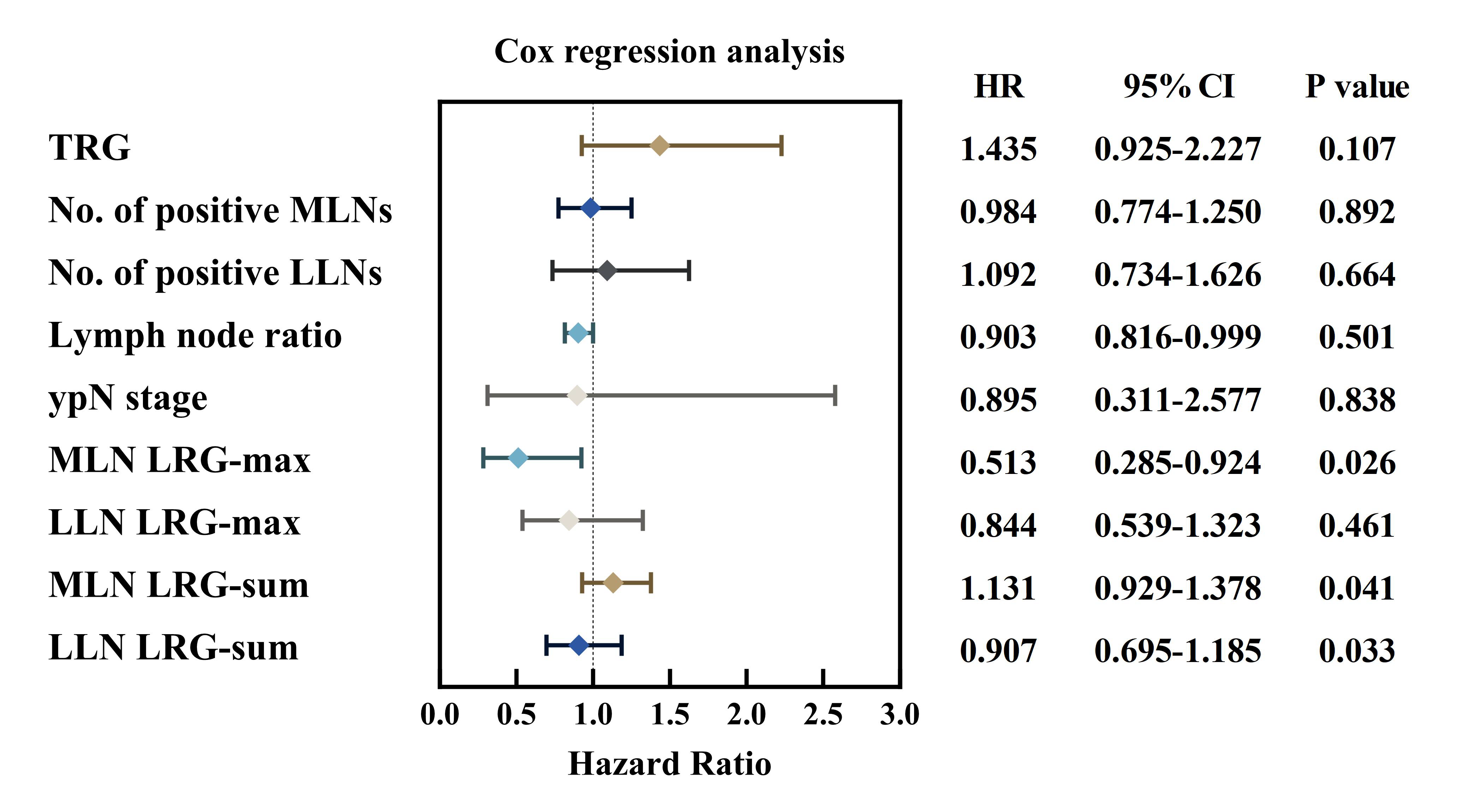
**

**Supplements Tables**

**Table S1 Distribution and Changes of MLNs in Regions and Size Categories Pre and Post-NCRT.**

| No. (%) | Pre-nCRT | Post-nCRT | Disappearance | Shrinkage | Stable | Progressive |
| --- | --- | --- | --- | --- | --- | --- |
| Total Mesorectal | 811 | 546 | 265 (32.7) | 389 (48) | 151 (18.6) | 6 (0.7) |
| 3-5mm | 476 (58.7) | 279 (51.1) | 197 (41.4) | 190 (39.9) | 83 (17.4) | 6 (1.3) |
| 5-7mm | 220 (27.1) | 166 (30.4) | 54 (24.5) | 131 (59.5) | 35 (16.0) | 0 (0) |
| ≥7mm | 115 (14.2) | 101 (18.5) | 14 (12.2) | 68 (59.1) | 33 (28.7) | 0 (0) |
| Above the peritoneal reflection | 547 (67.4) | 374 (68.5) | 173 (31.6) | 268 (49) | 101 (18.5) | 5 (0.9) |
| Below the peritoneal reflection | 264 (32.6) | 172 (31.5) | 92 (34.8) | 121 (45.8) | 50 (18.9) | 1 (0.5) |

**Table S2 Regression Rates of MLNs in Different Sizes and Regions Post- NCRT.**

| SA regression rate | Above the peritoneal reflection | Below the peritoneal reflection | P |
| --- | --- | --- | --- |
| Total | 0.588±0.331 | 0.631±0.338 | 0.546 |
| 3-5mm | 0.614±0.356 | 0.668±0.338 | 0.351 |
| 5-7mm | 0.588±0.289 | 0.565±0.303 | 0.488 |
| ≥7mm | 0.493±0.289 | 0.420±0.338 | 0.121 |
| SA: short-axis. |  |  |  |

**Table S3 Distribution and Changes of LLNs in Regions and Size Categories Pre and Post-NCRT.**

| No. (%) | Pre-nCRT | Post-nCRT | Disappearance | Shrinkage | Stable | Progressive |
| --- | --- | --- | --- | --- | --- | --- |
| Total Lateral | 993 | 969 | 24 (2.4) | 265 (26.7) | 679 (68.4) | 25 (2.5) |
| 3-5mm | 690 (69.5) | 674 (69.6) | 16 (2.3) | 140 (20.3) | 514 (74.5) | 20 (2.9) |
| 5-7mm | 175 (21.5) | 169 (17.4) | 6 (3.4) | 64 (36.6) | 103 (58.9) | 2 (1.1) |
| ≥7mm | 128 (9) | 126 (13) | 2 (1.6) | 61 (47.7) | 62 (48.4) | 3 (2.3) |
| Common iliac | 220 (22.2) | 210 (21.7) | 10 (4.5) | 31 (14.1) | 172 (78.2) | 7 (3.2) |
| External iliac | 126 (12.7) | 126 (13) | 0 (0) | 21 (16.7) | 102 (81) | 3 (2.4) |
| Proximal internal iliac | 79 (8) | 77 (7.9) | 2 (2.5) | 25 (31.6) | 48 (60.8) | 4 (5.1) |
| Distal internal iliac | 73 (7.4) | 71 (7.4) | 2 (2.7) | 33 (45.2) | 36 (49.3) | 2 (2.7) |
| Extended distal internal iliac | 11 (1.1) | 11 (1.1) | 0 (0) | 3 (27.3) | 8 (72.7) | 0 (0) |
| Obturator cranial | 347 (34.9) | 345 (35.6) | 2 (0.6) | 87 (25.1) | 253 (72.9) | 5 (1.4) |
| Obturator caudal | 137 (13.8) | 129 (13.3) | 8 (5.8) | 65 (47.4) | 60 (43.8) | 4 (2.9) |

**Table S4 Regression Rates of LLNs in Different Sizes and Regions Post- NCRT.**

| SA regression rate | Common iliac | External iliac | Internal iliac | Obturator | P |
| --- | --- | --- | --- | --- | --- |
| Total | 0.172±0.265 | 0.149±0.173 | 0.244±0.264 | 0.239±0.234 | <0.001 |
| 3-5mm | 0.123±0.236 | 0.134±0.170 | 0.187±0.257 | 0.205±0.226 | 0.001 |
| 5-7mm | 0.291±0.323 | 0.183±0.206 | 0.317±0.192 | 0.289±0.215 | 0.133 |
| ≥7mm | 0.389±0.207 | 0.159±0.141 | 0.364±0.330 | 0.371±0.241 | 0.003 |

**Table S5. LRG-Related Characteristics of 26 Patients with Both MLN and LLN Metastases.**

| Characteristics | MLN | LLN |
| --- | --- | --- |
| Retrieved lymph nodes | 215 | 224 |
| Positive lymph nodes | 80 | 52 |
| LRG-max (mean rank) | 21.37 | 31.63 |
| Score 1 | 3 (11.5) | 4 (15.4) |
| Score 2 | 10 (38.5) | 1 (3.8) |
| Score 3 | 5 (19.2) | 3 (11.5) |
| Score 4 | 7 (29.6) | 12 (46.2) |
| Score 5 | 1 (3.8) | 6 (23.1) |
| LRG-sum | 7.54±7.58 | 7.04±6.67 |
| LRG-ratio | 2.47±0.96 | 3.45±1.29 |
| Abbreviations: MLN, mesorectal lymph nodes; LLN, lateral pelvic lymph nodes; LRG, lymph node regression grade. | | |

**Table S6. Survival outcome between three LRG-sum groups.**

| Variable | MLN-LRGsum | | |  | LLN-LRGsum | | |
| --- | --- | --- | --- | --- | --- | --- | --- |
|  | low | middle | high |  | low | middle | high |
| 3-year OS rate | 84.20% | 62.10% | 38.10% |  | 90.20% | 58.80% | 54.10% |
| 3-year DFS rate | 70.90% | 58.20% | 22.00% |  | 78.20% | 44.80% | 29.00% |
| 5-year OS rate | 77.40% | 62.10% | 25.40% |  | 80.90% | 58.80% | 27.10% |
| 5-year DFS rate | 65.60% | 58.20% | 22.00% |  | 75.60% | 29.90% | 29.00% |

**Table S7. Cox regression analysis of factors for disease-free survival.**

| Characteristics | Univariate analysis | | |  | Cox regression analysis | | |
| --- | --- | --- | --- | --- | --- | --- | --- |
|  | HR | 95% CI | P value |  | HR | 95% CI | P value |
| Gender | 0.614 | 0.316-1.190 | 0.148 |  |  |  |  |
| Age | 1.005 | 0.975-1.035 | 0.759 |  |  |  |  |
| BMI | 0.964 | 0.923-1.077 | 0.321 |  |  |  |  |
| Distance from the anal verge | 0.740 | 0.610-0.897 | 0.265 |  |  |  |  |
| Preoperative CEA level | 1.006 | 0.993-1.019 | 0.358 |  |  |  |  |
| Tumor diameter | 1.037 | 0.877-1.225 | 0.672 |  |  |  |  |
| Lymphovascular invasion | 1.679 | 0.565-4.735 | 0.327 |  |  |  |  |
| Perineural invasion | 1.805 | 0.917-3.553 | 0.087 |  |  |  |  |
| CRM | 0.390 | 0.054-2.842 | 0.353 |  |  |  |  |
| Lymph node ratio | 0.901 | 0.823-0.987 | < 0.001 |  | 0.903 | 0.816-0.999 | 0.501 |
| ypT stage | 1.073 | 0.390-2.952 | 0.366 |  |  |  |  |
| ypN stage | 2.949 | 1.474-5.900 | 0.001 |  | 0.895 | 0.311-2.577 | 0.838 |
| Tumor regression grade | 1.584 | 1.063-2.359 | 0.024 |  | 1.435 | 0.925-2.227 | 0.107 |
| No. of retrieved MLNs | 1.018 | 0.975-1.063 | 0.424 |  |  |  |  |
| No. of positive MLNs | 1.150 | 1.029-1.284 | 0.014 |  | 0.984 | 0.774-1.250 | 0.892 |
| No. of retrieved LLNs | 0.953 | 0.904-1.004 | 0.073 |  |  |  |  |
| No. of positive LLNs | 1.513 | 1.252-1.827 | < 0.001 |  | 1.092 | 0.734-1.626 | 0.664 |
| MLN LRG-max | 1.305 | 1.057-1.610 | 0.013 |  | 0.513 | 0.285-0.924 | 0.026 |
| LLN LRG-max | 1.370 | 1.173-1.601 | < 0.001 |  | 0.844 | 0.539-1.323 | 0.461 |
| MLN LRG-sum | 1.819 | 1.215-2.722 | 0.004 |  | 1.131 | 0.929-1.378 | 0.041 |
| LLN LRG-sum | 2.234 | 1.605-3.395 | < 0.001 |  | 0.907 | 0.695-1.185 | 0.033 |
| MLN LRG-ratio | 1.295 | 1.022-1.641 | 0.032 |  |  |  |  |
| LLN LRG-ratio | 1.370 | 1.173-1.599 | < 0.001 |  |  |  |  |
| Abbreviations: BMI, body mass index; CEA, carcinoembryonic antigen; CRM, circumferential resection margin; MLN, mesorectal lymph nodes; LLN, lateral pelvic lymph nodes. | | | | | | | |
